# Positron emission tomography measurement of brain MAO-B inhibition in patients with Alzheimer’s disease and elderly controls after oral administration of sembragiline

**DOI:** 10.1007/s00259-016-3510-6

**Published:** 2016-09-16

**Authors:** Stefan Sturm, Anton Forsberg, Stephane Nave, Per Stenkrona, Nicholas Seneca, Andrea Varrone, Robert A. Comley, Patrik Fazio, Candice Jamois, Ryuji Nakao, Zbigniew Ejduk, Nabil Al-Tawil, Ulrika Akenine, Christer Halldin, Niels Andreasen, Benedicte Ricci

**Affiliations:** 1 Roche Innovation Center Basel, Roche Pharmaceutical Research and Early Development, Grenzacherstrasse 124, Basel, Switzerland; 2 Department of Clinical Neuroscience, Centre for Psychiatric Research, Karolinska Institutet, Stockholm, Sweden; 3 AstraZeneca Translational Science Center, Stockholm, Sweden; 4 Internal Disease and Gastroenterology, Miedzyleski Specialistic Hospital, Warsaw, Poland; 5 Karolinska Trial Alliance Phase 1 Unit, Karolinska University Hospital, Stockholm, Sweden; 6 Karolinska Institutet Alzheimer Disease Research Centre and Clinical Trial Unit, Geriatric Clinic, Karolinska University Hospital, Huddinge, Sweden; 7 AbbVie, North Chicago, IL USA; 8 F. Hoffmann-La Roche Ltd, Grenzacherstrasse 124, 4070 Basel, Switzerland

**Keywords:** Monoamine oxidase inhibitor, MAO-B, Sembragiline, Alzheimer’s disease, Positron emission tomography

## Abstract

**Purpose:**

In Alzheimer’s disease (AD), increased metabolism of monoamines by monoamine oxidase type B (MAO-B) leads to the production of toxic reactive oxygen species (ROS), which are thought to contribute to disease pathogenesis. Inhibition of the MAO-B enzyme may restore brain levels of monoaminergic neurotransmitters, reduce the formation of toxic ROS and reduce neuroinflammation (reactive astrocytosis), potentially leading to neuroprotection. Sembragiline (also referred as RO4602522, RG1577 and EVT 302 in previous communications) is a potent, selective and reversible inhibitor of MAO-B developed as a potential treatment for AD.

**Methods:**

This study assessed the relationship between plasma concentration of sembragiline and brain MAO-B inhibition in patients with AD and in healthy elderly control (EC) subjects. Positron emission tomography (PET) scans using [^11^C]-_L_-deprenyl-D_2_ radiotracer were performed in ten patients with AD and six EC subjects, who received sembragiline each day for 6–15 days.

**Results:**

At steady state, the relationship between sembragiline plasma concentration and MAO-B inhibition resulted in an E_max_ of ∼80–90 % across brain regions of interest and in an EC_50_ of 1–2 ng/mL. Data in patients with AD and EC subjects showed that near-maximal inhibition of brain MAO-B was achieved with 1 mg sembragiline daily, regardless of the population, whereas lower doses resulted in lower and variable brain MAO-B inhibition.

**Conclusions:**

This PET study confirmed that daily treatment of at least 1 mg sembragiline resulted in near-maximal inhibition of brain MAO-B enzyme in patients with AD.

**Electronic supplementary material:**

The online version of this article (doi:10.1007/s00259-016-3510-6) contains supplementary material, which is available to authorized users.

## Introduction

Expression of brain monoamine oxidase type B (MAO-B) increases with age in healthy subjects [1, 2], and is further increased in patients with Alzheimer’s disease (AD) [3, 4]. The brain areas with the greatest increase of MAO-B in patients with AD, ranging from 40 to 60 %, are also known to contain high densities of senile plaques and tangles (temporal and parietal cortices and hippocampus) [5]. Moreover, immunohistochemical and autoradiographic studies in AD post-mortem brain tissue have shown that MAO-B is highly expressed in reactive astrocytes (gliosis) surrounding amyloid plaques [6].

This up-regulation of MAO-B appears from an early stage, and the increased activity is maintained throughout the course of the disease [7], suggesting that MAO-B may contribute to neurodegenerative processes by increasing oxidative stress in at least two ways. Deamination of dopamine and other amines by MAO-B leads to the formation of hydrogen peroxide that, in the presence of trace amounts of metals, can be converted into highly toxic hydroxyl radicals. Such radicals react avidly with polyunsaturated fats in neuronal membranes, initiating lipid peroxidation and cell death [8]. Secondly, increases in MAO-B may also contribute to the reduced neurotransmitter levels in the brains of AD patients which may lead to the neuropsychological symptoms that are common in AD [6].

In humans, MAO-B preferentially deaminates dopamine and phenylethylamine and has been suggested to participate in the catabolism of noradrenaline [8]. In addition, MAO-B is highly expressed in serotonin-containing neurons such as those in the raphe nucleus. Dopamine and serotonin [9] levels are reported to be decreased in the brains of individuals with AD [10]. Thus, inhibition of MAO-B enzymatic activity is expected to produce beneficial effects in AD by a dual mechanism, namely, by reducing neurodegenerative processes due to oxidative stress and by re-establishing a physiological tonus of monoaminergic neurotransmission.

Additional evidence for the efficacy of MAO-B inhibitors (MAO-Bis) in the treatment of AD comes from clinical trials with the MAO-Bi selegiline. The results of a 2-year trial in patients with moderate AD suggest that selegiline may delay functional deterioration and disease progression [11]. In addition, lazabemide, a selective MAO-Bi developed by Roche but not continued due to hepatotoxic liability, showed a significant improvement in cognition and functionality in patients with AD at doses previously shown in a positron emission tomography (PET) study with healthy volunteers to provide full blockade of the enzyme [12].

Sembragiline is a potent, selective and slowly reversible MAO-Bi that has a novel chemical structure different from that of lazabemide and selegiline. Based on MAO enzymatic activity and displacement assays, the observed IC_50_ value from in vitro affinity assays of the MAO-B enzyme is 5–6 nM [13]. This is approximately 600-fold more potent in inhibiting MAO-B than MAO-A, and can thus achieve complete MAO-B inhibition without affecting the activity of MAO-A, as well as a wide variety of receptors, ion channels or other enzymes.

Approximately 1040 subjects have participated in phase 1 and 2 sembragiline studies for smoking cessation and moderate AD dementia [14]. In these studies, sembragiline was found to be safe and well tolerated. At low doses, the pharmacokinetics (PK) of sembragiline are less than dose-proportional, but after administration of approximately 5 mg of sembragiline, the PK become linear, dose-proportional and time-invariant, due to saturation of capacity-limited binding to the peripheral enzyme receptor. In steady-state conditions, sembragiline is slowly eliminated from the plasma, with a terminal half-life of about 50–65 h in elderly subjects.

PET imaging studies in healthy young men confirmed that sembragiline crosses the blood–brain barrier and inhibits brain MAO-B enzyme activity. A dose-related increase in brain MAO-B inhibition was demonstrated in young healthy subjects who received single doses of 5 and 15 mg sembragiline. Doses of 5 mg sembragiline, administered daily for 2–10 days, resulted in 52–78 % and 73–87 % inhibition in cortical and subcortical regions, respectively (Roche data on file). At a dose of 15 mg sembragiline, 68–74 % and 83–86 % inhibition of brain MAO-B was observed in cortical and subcortical regions, respectively; selegiline 20 mg, which was used as active comparator in the study, provided mean inhibition levels of 46–79 % for the different brain regions. Based on simulations with a population PK–enzyme inhibition model developed with data from 36 young healthy volunteers, a daily dose of 1 mg sembragiline is anticipated to reach near full MAO-B inhibition in most patients with AD, assuming no relevant age or disease effect on the PK–MAO-B inhibition relationship (Roche data on file).

The present repeated-dose PET study was undertaken to confirm near-complete MAO-B enzyme inhibition in the brain at the doses tested in a phase 2 clinical trial in patients with moderate AD, registered at ClinicalTrials.gov (NCT01677754). The objectives of this PET study were to determine the effects of varying multiple oral doses of sembragiline at steady state on brain MAO-B enzyme activity in patients with mild-to-moderate AD using PET imaging with [^11^C]-_L_-deprenyl-D_2_, and to characterise the sembragiline plasma–brain MAO-B inhibition relationship in patients with AD compared to healthy age-matched controls.

## Material and methods

The study was approved by the Regional Ethical Review Board in Stockholm, Sweden, the Radiation Safety Committee at the Karolinska University Hospital in Solna (Stockholm, Sweden) and the Medical Product Agency in Sweden. Patients with AD and healthy elderly controls (EC) were enroled in Karolinska Hospital at Huddinge, Sweden.

## Study design

In this multicentre, open-label, parallel-group, multiple-dose study, sembragiline was administered orally once daily for 6–15 days, depending on the dose group, to reach PK and enzyme inhibition steady-state conditions, and to allow for flexibility in PET scan scheduling. The first dose was administered at the study centre under the supervision of study personnel. On subsequent study days, treatment compliance was assured by intake logs and tablet counting.

At baseline, each subject underwent a PET scan using [^11^C]-_L_-deprenyl-D_2_ to measure MAO-B enzyme activity in the brain prior to their first dose of allocated medication. The second scan was performed at trough concentrations at steady state prior to the last drug administration. Before each scan, a cannula was inserted into a radial artery and another into an antecubital vein. A sterile phosphate buffer (pH = 7.4) containing radioligand was injected as a bolus over several seconds into the cubital vein.

## Subjects: inclusion, exclusion criteria

Healthy EC subjects with no evidence of AD or other cognitive impairment and patients with AD aged 50–80 years were enroled in the study. Informed consent was obtained from all EC subjects and patients with AD, co-signed by the patient’s closest relative and, for patients who were incapable of giving informed consent, a legally authorised representative.

Patients with probable AD based on the National Institute of Neurological and Communicative Disorders and Stroke and the Alzheimer’s Disease and Related Disorders Association (NINCDS/ADRDA) and the *Diagnostic and Statistical Manual of Mental Disorders*, 4th Edition (DSM-IV) criteria, having a Mini-Mental State Exam (MMSE) score of 17–26 inclusive, and a modified Hachinski Ischemia Scale score ≤4 were enroled in the study. A neuroimaging evaluation of the brain by magnetic resonance imaging (MRI) supported the diagnosis of AD, with no evidence of focal disease to account for dementia or MRI exclusion criteria.

Background AD therapy of anticholinesterase inhibitors (donepezil, galantamine, rivastigmine), antidepressants (citalopram, paroxetine, sertraline), antipsychotics (risperidone, quetiapine), and memantine were permitted, provided that they were maintained on a stable dosage regimen for at least 1 month before day 1 of the study. Further benzodiazepines with a short half-life (e.g. lorazepam), zolpidem, zopiclone, eszopiclone and trazodone for insomnia were permitted in patients with AD. Occasional use of oxazepam or lorazepam for anxiety due to MRI scan was permitted in all subjects.

EC subjects showed no clinically relevant findings on physical examination, vital signs, electrocardiography (ECG) or routine laboratory tests, and no suspicion of cognitive impairment or early dementia on the Rey Auditory Verbal Learning Test, MMSE, copying a cube and cross shown on paper, and drawing a time point on a clock. EC subjects were excluded if they had a family history of AD in first- or second-degree relatives <75 years of age. All subjects were instructed to follow a low-tyramine diet.

Exclusion criteria for patients with AD and EC subjects included prior exposure to ionising radiation or radioisotope for research that would exceed the local yearly radiation dose exposure, and implants or ferromagnetic foreign bodies that would present a risk during MRI.

## Study treatment

For dosing with target doses <1 mg, a loading dose of 5 mg was administered on day 1 to saturate the capacity-limited binding of sembragiline, allowing achievement of steady-state levels (PK and pharmacodynamic [PD]) within 7–14 days of dosing.

Group 1 was composed of three patients with AD and three EC subjects who received 1 mg sembragiline daily for 2 weeks. This dose regimen was expected to provide enzyme inhibition levels in the range of 80 % averaged across brain regions of interest (ROIs). A loading dose of 5 mg was to be administered on day 1 of treatment, followed by daily administration of target doses for doses <1 mg.

Group 2 was composed of three patients with AD and three EC subjects who received a loading dose of 5 mg on day 1 and doses of 0.2 mg/day for the following 6 days. This dose regimen was expected to provide enzyme inhibition levels in the range of 60–70 % averaged across brain ROIs.

Group 3 was composed of two patients with AD who received a dose of 5 mg of sembragiline in order to better characterise the plateau of enzyme inhibition in patients with AD.

Group 4 was composed of two patients with AD who received a 5-mg loading dose on day 1, followed by 0.1 mg of sembragiline for 13–16 days, to explore enzyme inhibition in lower concentration ranges. Male subjects were selected for group 4 in order to reduce the gender imbalance between the patients with AD and the EC subjects.

## Safety assessments

The safety of sembragiline was assessed by monitoring vital signs, laboratory tests and adverse events (AEs), and by evaluating the risk of suicide using the Columbia Suicide Severity Rating Scale.

## Synthesis/preparation of [^11^C]-_L_-deprenyl-D_2_

[^11^C]-_L_-deprenyl-D_2_ was prepared by *N*-methylation of the desmethyl _L_-deprenyl-D_2_ precursor using [^11^C]methyl triflate at room temperature [15]. The radiochemical yield was 40–50 %, and the radiochemical purity was >95 %. The entire synthesis was performed using a completely automated commercial radiochemistry module (Scansys PET chemistry module; Scansys Laboratorieteknik, Denmark).

## MRI

All subjects underwent MRI, performed on a 1.5-T Siemens MAGNETOM Avanto scanner at Medicinsk Röntgen, Odenplan, Stockholm, Sweden. T1-weighted MR images were obtained for each individual and used for grey and white matter segmentation and delineation of ROIs using the Anatomical Automatic Labeling (AAL) template [16].

The MR image was segmented into grey and white matter, and cerebrospinal fluid segments, using the SPM5 segmentation algorithm in MATLAB (Wellcome Trust Centre for Neuroimaging, London, UK; The MathWorks, Inc., Natick, MA, USA). ROIs encompassing the lateral frontal cortex, medial frontal cortex, lateral temporal cortex, and lateral parietal cortex, lateral occipital cortex, hippocampus, putamen, thalamus, cerebellum and whole brain were drawn on the co-registered MR image and mapped onto the PET images, and time–activity curves were extracted.

## PET scan procedures

A transmission scan was performed using three rotating ^68^Ge rod sources. [^11^C]-L-Deprenyl-D_2_ was injected intravenously as a bolus, and emission data were acquired over 63 min using the ECAT EXACT high-resolution system (Siemens Medical Solutions, Malvern, PA, USA). The injected radioactivity was targeted to approximately 300 MBq. The injected mass of deprenyl was to be ≤100 μg, and ≥50–100-fold lower than the typical administered dose of 5–10 mg deprenyl to patients with Parkinson’s disease. The framing was as follows: 9 frames x 10 s, 2 frames x 15 s, 3 frames x 20 s, 4 frames x 30 s, 4 frames x 60 s, 4 frames x 180 s, 7 frames x 360 s. Images were reconstructed using the standard filtered back-projection, with a 2-mm Hanning filter, a zoom factor of 2.17, and a 128 × 128 matrix, and were corrected for attenuation and scatter [17]. A summation PET image was created for each subject and PET session by averaging across all frames. Time–activity curves were then extracted from selected ROIs.

## Arterial blood sampling

Arterial blood samples were used for measurement of radioactivity in whole blood and plasma, as well as to determine the parent fraction of the radioligand, using a reversed-phase high-performance liquid chromatography method (Karolinska University Hospital, Solna, Stockholm, Sweden). A catheter was inserted in the radial artery, and arterial blood was collected continuously for 10 min using an automated blood sampling system at a speed of 5 mL/min. Discrete blood samples were drawn approximately 10, 15, 20, 30, 45 and 60 min after each injection of [^11^C]-_L_-deprenyl-D_2_.

## Quantitation of [^11^C]-_L_-deprenyl-D_2_ binding

Time–activity data were analysed as previously described [18, 19]. Briefly, [^11^C]-_L_-deprenyl-D_2_ was quantified by the two-tissue compartment model with three rate constants (*K*_1_, *k*_2_, *k*_3_) using the PMOD 3.3 software package (PMOD Group, Zurich, Switzerland), where the tissue compartments represent the free plus non-specifically bound and specifically bound radiotracer. The outcome measure λ*k*_3_ was calculated as (*K*_1_/*k*_2_)*k_3_, using a non-linear approach, and was used as an estimate for specific binding to MAO-B that was independent of perfusion. The parameter λk3 (where λ = K1/k2) was used as an estimate for specific binding to MAO-B, as it was found to reflect the regional enzyme concentration more accurately than k3 alone [19]. This is because λk3 is independent of perfusion and has better reproducibility than k3 alone [20].

Inhibition of MAO-B in the brain by sembragiline from regional λ*k*_3_ was calculated as follows:

$$ Inhibition=100\ \%\ x\ \left(\left|\lambda {k}_3 drug - \lambda {k}_3 baseline\right|/\lambda {k}_3 baseline\right) $$

Here, “drug” refers to scans after drug administration.

## Assessment of PK

Blood samples were collected for sembragiline plasma concentrations and analysed with a validated liquid chromatography–tandem mass spectrometry method (Covance Inc., Harrogate, North Yorkshire, UK). Plasma concentrations of sembragiline were measured just prior to the second PET scan.

## Sembragiline plasma concentration–MAO-B enzyme inhibition relationship

Pre-scan plasma concentrations were used for the analysis of the relationship between MAO-B enzyme inhibition and steady-state sembragiline plasma concentration, and were characterised using a simple E_max_ model for each brain ROI:

$$ Occ\ \left(\%\right)={E}_{max}*{C}_p/\left({C}_p+E{C}_{50}\right) $$

,where C_p_ = 0 ng/mL at baseline and C_p_ = infinity (ng/mL) at E_max_; and where C_p_ is sembragiline plasma concentration (ng/mL), E_max_ is maximum MAO-B enzyme inhibition (%) and EC_50_ is sembragiline concentration (ng/mL) at half-maximal inhibition.

The PK/PD analysis was performed using Phoenix WinNonlin software version 6.2 (Pharsight Corporation, Mountain View, CA, USA).

## Results

Seventeen subjects (11 patients with AD and six EC subjects) were enroled in the study. One enroled subject, a patient with AD assigned to group 1, withdrew from the study because it was not possible to perform arterial cannulation, and thus no baseline PET scan was performed and no study treatment was administered.

The demographics of the 16 patients who received sembragiline are summarised in Supplementary Table 1. Patients with AD were on stable background AD therapy with galantamine, donepezil, memantine, or a combination of galantamine and memantine. All subjects were non-smokers. There was an imbalance in gender between the patients with AD and the healthy EC subjects. The majority (80 %) of patients with AD were female, whereas the majority (83 %) of EC subjects were male. Healthy control subjects (70.5 years) were on average slightly older than the AD patients (65.0 years).

## Treatment compliance

All EC subjects documented daily drug intake and returned the expected number of tablets. Two patients with AD each missed one dose. Three patients with AD returned fewer tablets than expected at the end of the study.

## Safety

Sembragiline treatment was well tolerated in patients with AD and EC subjects. No AE led to withdrawal from the study, and all subjects completed their assigned treatment. In total, 11 of 20 AEs were reported as drug-related. Four of six EC subjects experienced 13 AEs during the study, and seven AEs were reported in three of ten patients with AD. Most AEs were of mild intensity. There was no trend for increased AEs with dose and no trend for body system-specific AEs. The most frequently observed AE was diarrhoea, which occurred in three subjects. There were no clinically significant changes in laboratory parameters, vital signs or ECG measurements.

## Sembragiline plasma concentrations

Mean plasma sembragiline concentrations taken prior to the second PET scan and last dose administration are summarised in Supplementary Table 2. With a loading dose of 5 mg sembragiline for low doses (<1 mg) or multiple doses for higher doses (≥1 mg), steady-state conditions were reached within 14 days and at the time of the second PET scan. Plasma concentrations at the time of PET scanning were in the expected range, based on simulations with a population-PK model previously developed in healthy volunteers (Roche data on file). A trend toward higher exposure of sembragiline in patients with AD (80 % female) compared with EC subjects was suggested, where 83 % of enroled subjects were male. A gender difference might explain the difference in PK between the two populations.

## Dosage of the [^11^C]-L-deprenyl-D2 tracer

The injected radioactivity and injected mass were 310 ± 35 MBq and 280 ± 37 MBq, and 0.28 ± 0.16 μg and 0.55 ± 0.60 μg, for EC subjects and AD patients, respectively.

A maximum injected mass of 5 μg was considered the threshold, and injected doses were well below this value, ensuring there was no effect on inhibition.

Injected radioactivity and total dose of the radiotracer per treatment group, scan and population are listed in Supplementary Table 3.

## [^11^C]-_L_-Deprenyl-D_2_ binding in the brain

**Fig. 1:**
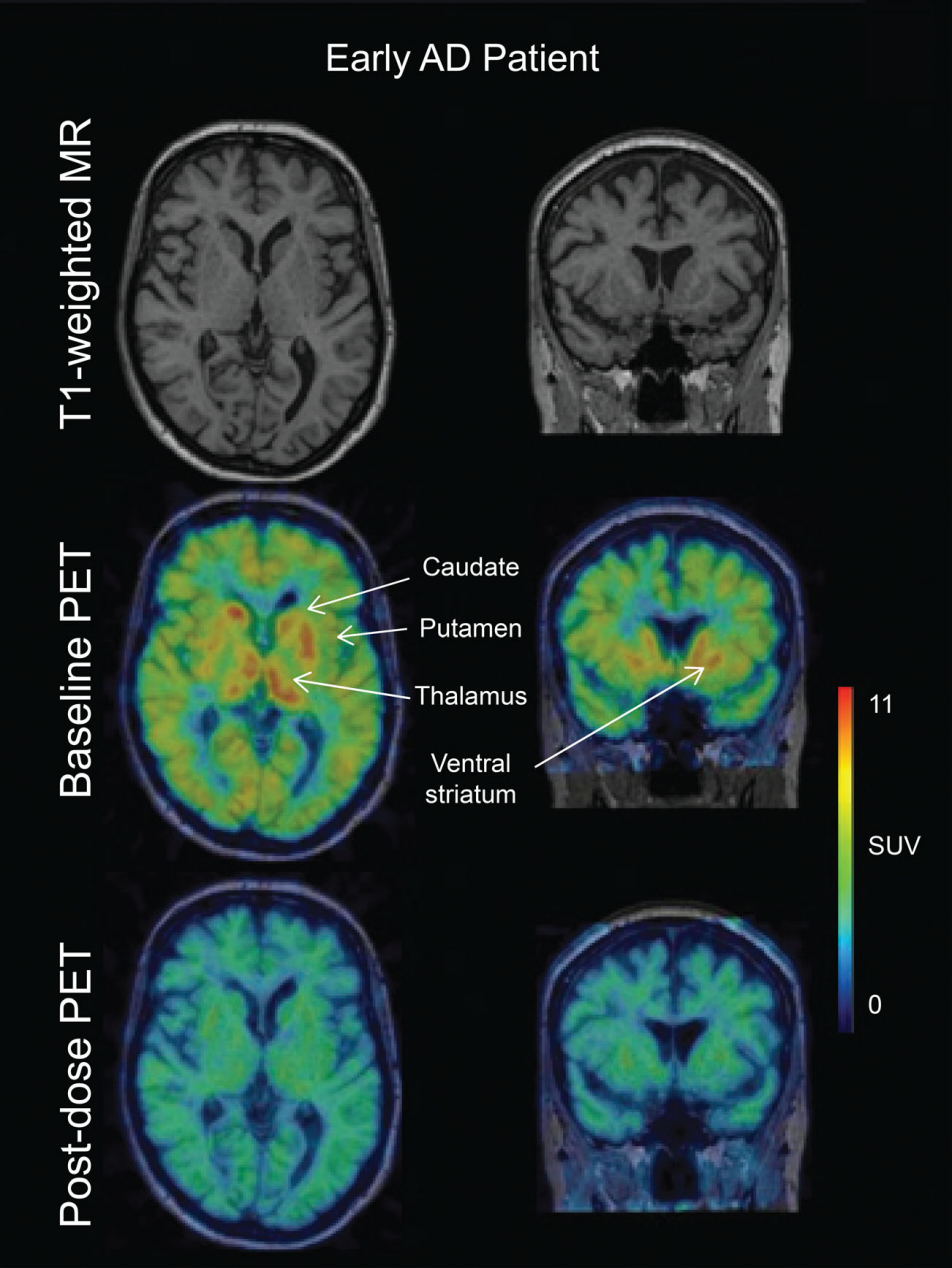
Visualization of MAO-B inhibition in the brain following treatment with 1 mg sembragiline once a day for 2 weeks. Axial, coronal and sagittal slices of [^11^C]-_L_-deprenyl-D_2_ PET images from a patient with AD in the 1-mg group are presented before and after sembragiline treatment

**Fig. 2:**
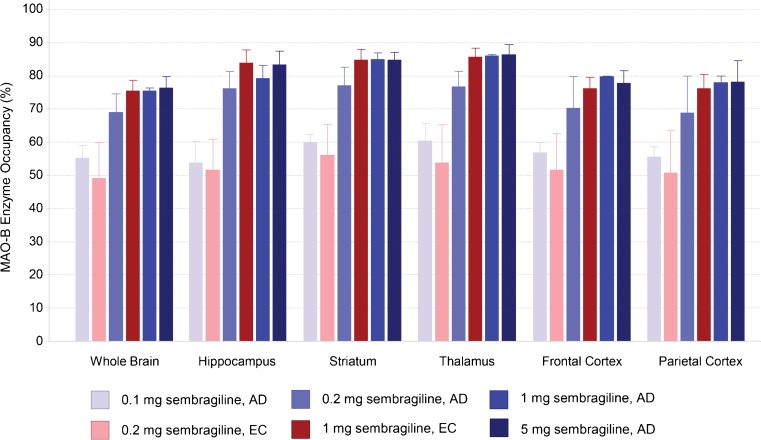
Regional brain MAO-B inhibition values (%) associated with the various drug regimens. *Error bars* represent standard deviations. Abbreviations: AD, patients with Alzheimer’s disease; EC, healthy elderly control; MAO-B, monoamine oxidase type B

λ*k*_3_ data representing specific binding of the [^11^C]-_L_-deprenyl-D_2_ tracer to MAO-B enzyme are summarised in Table 1. λ*k*_3_ values before and after treatment are presented. Baseline λ*k*_3_ values in patients with AD were similar to those observed in healthy EC subjects. In both patients with AD and EC subjects, baseline λ*k*_3_ values were highest in the striatum and lower in the cortical regions. Mean regional MAO-B inhibition levels induced by the different drug regimens are shown in Table 1 and visualised in Figs. 1 and 2.

**Table 1:** Regional [^11^C]-_L_-deprenyl-D_2_ λk3 values before and after sembragiline treatment and respective occupancy values induced by the drug regimens by population

		Whole brain	Hippocampus	Striatum	Thalamus	Frontal cortex	Parietal cortex
AD	Baseline	0.18 (0.16–0.21)	0.24 (0.20–0.27)	0.30 (0.27–0.33)	0.28 (0.21–0.34)	0.17 (0.14–0.19)	0.17 (0.15–0.19)
0.1 mg	Post-dose	0.08 (0.08–0.09)	0.11 (0.10–0.11)	0.12 (0.11–0.13)	0.11 (0.09–0.1)	0.07 (0.06–0.08)	0.07 (0.07–0.08)
N = 2	Occ, %	55.2 (52.5–57.9)	53.8 (49.2–58.4)	59.9 (58.3–61.6)	60.4 (56.7–64.1)	56.9 (54.8–59.0)	55.6 (53.4–57.7)
EC	Baseline	0.16 (0.15–0.16)	0.23 (0.22–0.24)	0.27 (0.26–0.28)	0.25 (0.24–0.27)	0.15 (0.15–0.16)	0.15 (0.14–0.16)
0.2 mg	Post-dose	0.08 (0.07–0.10)	0.11 (0.09–0.13)	0.12 (0.09–0.14)	0.12 (0.09–0.13)	0.07 (0.06–0.09)	0.07 (0.06–0.09)
N = 3	Occ, %	49.1 (37.5–58.8)	51.6 (42.3–60.9)	56.1 (47.9–66.3)	53.7 (44.5–66.6)	51.7 (41.9–63.5)	50.7 (37.2–62.3)
AD	Baseline	0.20 (0.18–0.24)	0.31 (0.26–0.36)	0.35 (0.29–0.45)	0.31 (0.26–0.36)	0.21 (0.18–0.26)	0.20 (0.17–0.25)
0.2 mg	Post-dose	0.06 (0.06–0.07)	0.07 (0.07–0.08)	0.08 (0.08–0.08)	0.07 (0.07–0.07)	0.06 (0.05–0.07)	0.06 (0.05–0.07)
N = 3	Occ, %	68.9 (64–75.2)	76.1 (70.7–81.1)	77.1 (72.5–83.3)	76.8 (71.7–80.6)	70.2 (60.4–79.4)	68.7 (57.1–79.3)
EC	Baseline	0.19 (0.19–0.20)	0.28 (0.25–0.31)	0.35 (0.33–0.38)	0.32 (0.29─0.36)	0.18 (0.17─0.19)	0.18 (0.15–0.20)
1 mg	Post-dose	0.05 (0.04–0.05)	0.04 (0.03–0.05)	0.05 (0.04–0.06)	0.05 (0.04–0.05)	0.04 (0.04–0.05)	0.04 (0.04–0.05)
N = 3	Occ, %	75.4 (73.2–79.2)	83.9 (79.7–87.6)	84.7 (81.3–87.6)	85.7 (82.9–88.2)	76.2 (72.7–79.3)	76.1 (71.3–79.9)
AD	Baseline	0.16 (0.14–0.19)	0.22 (0.19–0.28)	0.28 (0.24–0.33)	0.25 (0.20–0.27)	0.16 (0.12–0.22)	0.16 (0.11–0.22)
1 mg	Post-dose	0.04 (0.04–0.05)	0.05 (0.05–0.05)	0.04 (0.04–0.05)	0.04 (0.04–0.04)	0.04 (0.03–0.04)	0.04 (0.03–0.05)
N = 3, 2^a^	Occ, %	75.5 (74.9–76.2)	79.2 (76.3–82.1)	85.0 (83.6–86.4)	86.0 (85.7–86.3)	79.7 (79.6–79.9)	78.0 (76.7–79.3)
AD	Baseline	0.19 (0.18–0.19)	0.24 (0.24–0.24)	0.30 (0.29–0.31)	0.28 (0.21–0.34)	0.18 (0.16–0.19)	0.18 (0.17–0.19)
5 mg	Post-dose	0.04 (0.04–0.05)	0.04 (0.03–0.05)	0.05 (0.04–0.05)	0.11 (0.09–0.12)	0.04 (0.04–0.04)	0.04 (0.03–0.04)
N = 2	Occ, %	76.3 (73.8–78.7)	83.3 (80.3–86.3)	84.8 (83.3–86.4)	86.3 (84.2–88.5)	77.8 (75.2–80.4)	78.1 (73.5–82.7)
AD	Baseline	0.18 (0.14–0.24)	0.26 (0.19–0.36)	0.31 (0.24–0.45)	0.28 (0.20–0.36)	0.18 (0.12–0.26)	0.18 (0.11–0.25)
N = 10							
EC	Baseline	0.18 (0.15–0.20)	0.25 (0.22–0.31)	0.31 (0.26–0.48)	0.29 (0.24–0.36)	0.17 (0.15–0.19)	0.16 (0.14–0.20)
N = 10							

Values are means (range)

Abbreviations: AD, patients with Alzheimer’s disease; EC, healthy elderly control; 0.1 mg, loading dose of 5 mg on day 1 followed by 0.1 mg q.d. for days 2–15; 0.2 mg, loading dose of 5 mg on day 1 followed by 0.2 mg q.d. on days 2–7; 1 mg, 1 mg q.d. for 14 days; 5 mg, 5 mg q.d. for 6–9 days

^a^ N = 3 for baseline λk3 values and N = 2 for post-dose λk3 and occupancy values

MAO-B inhibition at trough following 1 mg sembragiline treatment was 75–86 % (mean values) across all brain ROIs and study populations, and was in the same range as that reported with the 5-mg dose in this study (mean values: 76–86 %). Doses of 0.1 mg and 0.2 mg were associated with lower and highly variable levels of inhibition. Across all treatment groups, the highest levels of inhibition were observed in subcortical regions such as the hippocampus, thalamus and striatum.

**Fig. 3:**
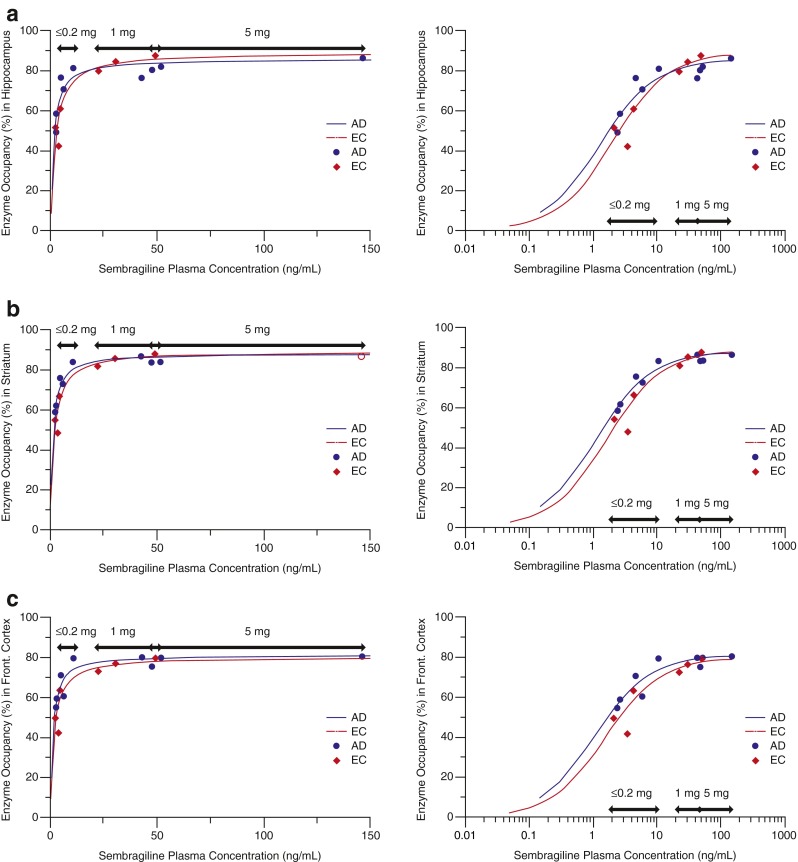
Relationship between pre-scan plasma concentration of sembragiline and MAO-B inhibition in patients with AD and EC subjects in steady-state conditions following sembragiline treatment. (**a**) hippocampus (**b**) striatum and (**c**) frontal cortex. Sembragiline was administered once daily p.o. over 6–15 days at doses of between 0.1 and 5 mg. At doses of 0.1 mg and 0.2 mg, a loading dose of 5 mg of sembragiline was administered. The *arrows* represent the range of pre-scan concentrations at given sembragiline doses

Plots of pre-scan sembragiline concentration versus MAO-B inhibition in steady-state conditions showed a sharp increase at low plasma concentrations to a plateau in MAO-B inhibition (Fig. 3). This was observed for both patients with AD and EC subjects. The plateau occurred at plasma concentrations of 20–50 ng/mL.

At steady state, the sembragiline plasma concentration–inhibition relationship could be described by a simple E_max_ model. Estimated E_max_ was 80–89 % and EC_50_ was 1–2 ng/mL across ROIs and for both populations (Table 2). Variability in PET data estimates was low to moderate, with a coefficient of variation (CV) below 40 % for all parameters. A population-PD model based on data from healthy volunteers and patients with AD yielded very similar E_max_ values, predicting median enzyme inhibition of >80 % for doses ≥1 mg in the hippocampus 24 h post-last dose (Roche data on file). Slightly lower EC_50_ values were estimated for patients with AD than for EC subjects.

**Table 2:** E_max_ and EC_50_ values of relationship between pre-scan plasma concentration of sembragiline and MAO-B occupancy of sembragiline in steady-state conditions by population and ROI

Population	Region of interest	E_max_, %	EC_50_, ng/mL
EC	Hippocampus	89.1 (CV%: 9.6)	2.05 (CV%: 33.4)
	Striatum	88.8 (CV%: 7.5)	1.67 (CV%: 29.7)
	Frontal cortex	80.1 (CV%: 8.9)	1.57 (CV% 36.2)
AD	Hippocampus	85.9 (CV%: 4.1)	1.29 (CV%: 21.5)
	Striatum	87.8 (CV%: 1.8)	1.11 (CV%: 10.7)
	Frontal cortex	81.1 (CV%: 3.2)	1.06 (CV%: 19.6)

Abbreviations: CV, coefficient of variation; EC_50_, concentration at half-maximal occupancy; E_max_ maximum MAO-B occupancy; EC, healthy elderly controls, AD, patients with Alzheimer’s disease; ROI, region of interest

Two patients with AD received concomitant sedative medication on an as-needed basis; one received 5 mg oxazepam and the other received 5 mg zopiclone. Data from these patients did not reveal any differences compared to other subjects with regard to sembragiline plasma concentrations or MAO-B inhibition. To the best of our knowledge, MAO-B is not a known target of oxazepam or zopiclone.

## Discussion

The purpose of the present imaging study was to determine the relationship between exposure to sembragiline and the inhibition of MAO-B enzyme activity in the brain after multiple oral doses in patients with AD. These data were used to assist in dose selection of the phase 2 sembragiline study in patients with moderate AD and in subsequent studies [21].

[^11^C]-_L_-Deprenyl-D_2_ was used to determine brain MAO-B inhibition of sembragiline. Previous imaging studies were performed with this radioligand for brain inhibition of MAO-Bis in healthy subjects [22], in elderly subjects in and patients with AD [23].

MAO-B inhibition following 1-mg treatment of sembragiline was 75–86 % (mean values) across the six brain ROIs (71–88 % overall) and study populations, and in the same range as those reported for 5-mg doses, indicating that plateau of inhibition had been reached at these doses. Doses of 0.1 and 0.2 mg were associated with lower and highly variable levels of inhibition. At a dose of 0.2 mg, women showed higher MAO-B inhibition than men, which is consistent with higher observed concentrations of sembragiline. However, similar MAO-B inhibition between men and women was observed after administration of a 1-mg dose, despite higher sembragiline concentrations in women. This suggests that for near-full MAO-B inhibition doses in the overall population, around 1 mg might be needed, and could not be achieved with lower doses (≤0.2 mg).

Across all treatment groups, a trend was observed for higher levels of inhibition in subcortical regions such as the hippocampus, thalamus and striatum than in cortical regions. This is consistent with simulated median enzyme inhibition with this compound based on population-PK model findings in healthy young volunteers (Roche data on file), with other MAO-Bi treatment in EC subjects [24], and with patients with AD [23]. These trends might be explained by non-specific binding of [^11^C]-_L_-deprenyl-D_2_, resulting in different binding capacity at baseline, or by differences in *K*_1_, and may be reflective of MAO-B activity, as the binding capacity was highest in the striatum and lower in the cortical regions in both patients with AD and EC subjects in the present study as well in others [23, 25].

In this study, baseline λ*k*_3_ values in patients with AD were similar to those observed in EC subjects, in contrast to previous reports where higher baseline [^11^C]-_L_-deprenyl-D_2_ binding was observed in patients with AD [3, 23]. However, the present analysis is limited by the overall small sample size and the slightly older EC population. When corrected for age, a slight trend for higher λ*k*_3_ in patients with AD was observed. MAO-B is reduced and inhibited in smokers [26]; however, as all subjects in this study were non-smokers, smoking status was not a confounding factor in the comparison of the two populations.

## Concentration–enzyme inhibition relationship

The mean steady-state inhibition values were in the expected range based on simulations with a preliminary PK enzyme inhibition model. Plots of MAO-B inhibition versus concentration showed a sharp increase at low concentrations, to a plateau in MAO-B inhibition. The relationship between plasma concentration and MAO-B inhibition could be well described with an E_max_ model. The estimated EC_50_ of 1–2 ng/mL was comparable to the observed IC_50_ (5–6 nM) value from in vitro affinity assays for the MAO-B enzyme (MAO enzymatic activity and displacement assays). PK/PD analysis per study population yielded very similar E_max_ and EC_50_ values for patients with AD and EC subjects. Slightly lower mean EC_50_ values for the different ROIs were estimated for patients with AD compared with EC subjects. However, this analysis is limited by the small sample size, and this finding might be explained by the variability in PK and inhibition data. These data provide a good understanding of the concentration–enzyme inhibition relationship and assist in dose selection for clinical efficacy studies. Regardless of the population, sembragiline concentrations around 20 ng/mL might be needed for near-complete MAO-B inhibition in the brain.

In conclusion, sembragiline treatment was well tolerated and resulted in near-complete inhibition of the brain MAO-B enzyme at 1 mg and 5 mg daily doses in patients with AD and EC subjects. At these doses, near-maximal inhibition of brain MAO-B was achieved regardless of the population. In the phase 2 trial, sembragiline at 1 and 5 mg daily failed on the primary efficacy outcome on cognition (Alzheimer’s Disease Assessment Scale–cognitive subscale (ADAS-Cog11) but showed a trend toward an effect on functioning and potential for an effect on neuropsychiatric symptoms compared with placebo. The lack of a cognitive effect in moderate AD is consistent with the effect of the MAO-B inhibitor, selegiline, which showed no long-term benefit on cognition in patients with moderate-to-severe AD [11]; rather, a treatment effect has been observed only in 4–6- or 8–17-week studies [27]. The effects of sembragiline are also not in accord with the results on cognition and functioning observed with the MAO-B inhibitor lazabemide. Together, these studies suggest that MAO-B inhibitors administered in addition to background therapy (i.e. acetylcholinesterase inhibitors, memantine) in patients with moderate AD may not add to the cognitive benefit.

## Electronic supplementary material

Below is the link to the electronic supplementary material.

ESM 1 (DOCX 18 kb)

## References

[1] Saura J, Andres N, Andrade C, Ojuel J, Eriksson K, Mahy N (1997). Biphasic and region-specific MAO-B response to aging in normal human brain. Neurobiol Aging.

[2] Fowler JS, Volkow ND, Wang GJ, Logan J, Pappas N, Shea C (1997). Age-related increases in brain monoamine oxidase B in living healthy human subjects. Neurobiol Aging.

[3] Reinikainen KJ, Paljarvi L, Halonen T, Malminen O, Kosma VM, Laakso M (1988). Dopaminergic system and monoamine oxidase-B activity in Alzheimer’s disease. Neurobiol Aging.

[4] Strolin Benedetti M, Dostert P (1989). Monoamine oxidase, brain ageing and degenerative diseases. Biochem Pharmacol.

[5] Nakamura S, Kawamata T, Akiguchi I, Kameyama M, Nakamura N, Kimura H (1990). Expression of monoamine oxidase B activity in astrocytes of senile plaques. Acta Neuropathol.

[6] Saura J, Luque JM, Cesura AM, Da Prada M, Chan-Palay V, Huber G (1994). Increased monoamine oxidase B activity in plaque-associated astrocytes of Alzheimer brains revealed by quantitative enzyme radioautography. Neuroscience.

[7] Kennedy BP, Ziegler MG, Alford M, Hansen LA, Thal LJ, Masliah E (2003). Early and persistent alterations in prefrontal cortex MAO A and B in Alzheimer’s disease. J Neural Transm.

[8] Riederer P, Danielczyk W, Grunblatt E (2004). Monoamine oxidase-B inhibition in Alzheimer’s disease. Neurotoxicology.

[9] Roth BL, Hanizavareh SM, Blum AE (2004). Serotonin receptors represent highly favorable molecular targets for cognitive enhancement in schizophrenia and other disorders. Psychopharmacology (Berl).

[10] Mitchell RA, Herrmann N, Lanctot KL (2011). The role of dopamine in symptoms and treatment of apathy in Alzheimer’s disease. CNS Neurosci Ther.

[11] Sano M, Ernesto C, Thomas RG, Klauber MR, Schafer K, Grundman M (1997). A controlled trial of selegiline, alpha-tocopherol, or both as treatment for Alzheimer’s disease. The Alzheimer’s Disease Cooperative Study. N Engl J Med.

[12] Magni G, Meibach RC (1999). Lazabemide for the long-term treatment of Alzheimer’s disease. Eur Neuropsychopharmacol.

[13] Borroni E, Wyler R, Messer J, Nave S, Cesura A (2013). Preclinical characterization of R04602522, a novel, selective, and orally active monoamine oxidase type B Inhibitor for the treatment of Alzheimer’s Disease. Alzheimers Dement.

[14] Berlin I, Hunneyball IM, Greiling D, Jones SP, Fuder H, Stahl HD (2012). A selective reversible monoamine oxidase B inhibitor in smoking cessation: effects on its own and in association with transdermal nicotine patch. Psychopharmacology (Berl).

[15] Nagren K, Muller L, Halldin C, Swahn CG, Lehikoinen P (1995). Improved synthesis of some commonly used PET radioligands by the use of [11C]methyl triflate. Nucl Med Biol.

[16] Tzourio-Mazoyer N, Landeau B, Papathanassiou D, Crivello F, Etard O, Delcroix N (2002). Automated anatomical labeling of activations in SPM using a macroscopic anatomical parcellation of the MNI MRI single-subject brain. Neuroimage.

[17] Wienhard K, Dahlbom M, Eriksson L, Michel C, Bruckbauer T, Pietrzyk U (1994). The ECAT EXACT HR: performance of a new high resolution positron scanner. J Comput Assist Tomogr.

[18] Bench CJ, Price GW, Lammertsma AA, Cremer JC, Luthra SK, Turton D (1991). Measurement of human cerebral monoamine oxidase type B (MAO-B) activity with positron emission tomography (PET): a dose ranging study with the reversible inhibitor Ro 19-6327. Eur J Clin Pharmacol.

[19] Fowler JS, Wang GJ, Logan J, Xie S, Volkow ND, MacGregor RR (1995). Selective reduction of radiotracer trapping by deuterium substitution: comparison of carbon-11-L-deprenyl and carbon-11-deprenyl-D2 for MAO B mapping. J Nucl Med.

[20] Logan J, Fowler JS, Volkow ND, Wang GJ, MacGregor RR, Shea C (2000). Reproducibility of repeated measures of deuterium substituted [11C]L-deprenyl ([11C]L-deprenyl-D2) binding in the human brain. Nucl Med Biol.

[21] Nave S, Doody RS, Boada M, Grimmer T, Savola J, Delmar P (2015). Sembragiline in moderate Alzheimer’s disease dementia: results of a phase 2 trial (MAyflOwer RoAD). J Prevent Alzheimer’s Dis.

[22] Fowler JS, Volkow ND, Logan J, Wang GJ, MacGregor RR, Schyler D (1994). Slow recovery of human brain MAO B after L-deprenyl (Selegeline) withdrawal. Synapse.

[23] Hirvonen J, Kailajarvi M, Haltia T, Koskimies S, Nagren K, Virsu P (2009). Assessment of MAO-B occupancy in the brain with PET and [11C]-L-deprenyl-D2: a dose-finding study with a novel MAO-B inhibitor, EVT 301. Clin Pharmacol Ther.

[24] Carter SF, Scholl M, Almkvist O, Wall A, Engler H, Langstrom B (2012). Evidence for astrocytosis in prodromal Alzheimer disease provided by 11C-deuterium-L-deprenyl: a multitracer PET paradigm combining 11C-Pittsburgh compound B and 18F-FDG. J Nucl Med.

[25] Fowler JS, MacGregor RR, Wolf AP, Arnett CD, Dewey SL, Schlyer D (1987). Mapping human brain monoamine oxidase A and B with 11C-labeled suicide inactivators and PET. Science.

[26] Fowler JS, Logan J, Wang GJ, Volkow ND (2003). Monoamine oxidase and cigarette smoking. Neurotoxicology.

[27] Birks J, Flicker L. Selegiline for Alzheimer’s disease. Cochrane Database Syst Rev. 2003;(1)(1):CD000442. 10.1002/14651858.CD000442 12535396

