# Supplementary material for: Positron emission tomography measurement of brain MAO-B inhibition in patients with Alzheimer’s disease and elderly controls after oral administration of sembragiline
Source: Eur J Nucl Med Mol Imaging. 2016 Sep 16;44(3):382–91. doi: 10.1007/s00259-016-3510-6 (PMC5281649; doi:10.1007/s00259-016-3510-6)
Supplement: Supplementary file 1 — (DOCX 18 kb) [file 259_2016_3510_MOESM1_ESM.docx]

**Supplementary Materials**

**Positron emission tomography measurement of brain MAO-B inhibition in patients with Alzheimer’s disease and elderly controls after oral administration of Sembragiline**

Stefan Sturm, PhD^1^*; Anton Forsberg, PhD^2^*; Stephane Nave, MD^1^; Per Stenkrona, MD^2^; Nicholas Seneca, PhD^3^; Andrea Varrone, MD, PhD^2^; Robert A. Comley, MSc^1†^; Patrik Fazio, PhD^2^; Candice Jamois, PharmD^1^; Ryuji Nakao, PhD^2^; Zbigniew Ejduk, MD, MBA^4^; Nabil Al-Tawil, MD, PhD^5^; Ulrika Akenine, RN^6^; Christer Halldin, PhD^2^; Niels Andreasen, MD, PhD^6^; Benedicte Ricci, PharmD, PhD^1^

1- Roche Innovation Center Basel, Roche Pharmaceutical Research and Early Development, Grenzacherstrasse 124, Basel, Switzerland;

2- Karolinska Institutet, Department of Clinical Neuroscience, Centre for Psychiatric Research, Stockholm, Sweden;

3- AstraZeneca Translational Science Center, Stockholm, Sweden;

4- Internal Disease and Gastroenterology, Miedzyleski Specialistic Hospital, Warsaw, Poland;

5- Karolinska Trial Alliance Phase 1 Unit, Karolinska University Hospital, Stockholm, Sweden;

6- Karolinska Institutet Alzheimer Disease Research Centre and Clinical Trial Unit, Geriatric Clinic, Karolinska University Hospital, Huddinge, Sweden;

*****These authors contributed equally

^†^Present address: AbbVie, North Chicago, IL, USA

**Corresponding author:
Name:** Stefan Sturm **Postal address:**F. Hoffmann-La Roche Ltd
Grenzacherstrasse 124
4070 Basel
Switzerland
**Telephone:** +41 61 687 52 64
**Email address:** stefan.sturm@roche.com

**Supplementary Tables**

**Supplementary Table 1**

**Demographic characteristics of EC subjects and patients with AD per treatment group and per population (means (SD))**

|  | **EC subjects** | | **AD patients** | | | |
| --- | --- | --- | --- | --- | --- | --- |
|  | 0.2 mg | 1 mg | 0.1 mg | 0.2 mg | 1 mg | 5 mg |
| N | 3 | 3 | 2 | 3 | 3 | 2 |
|  | 6 | | 10 | | | |
| Age | 64.3 (11.6) | 76.7 (3.2) | \| 61.5 (9.2) \| \| --- \| | 65.3 (8.3) | 65.7 (11.9) | 67.0 (9.9) |
|  | 70.5 (10.2) | | 65.0 (8.4) | | | |
| Male, N (%) | 3 (100) | 2 (66.7) | 2 (100) | 0 | 0 | 0 |
|  | 5 (83) | | 2 (20) | | | |
| Weight | 81.7 (5.0) | 88.0 (10.2) | \| 83.0 (1.4) \| \| --- \| | 62.3 (4.0) | 72.5 (21.6) | 76.0 (21.2) |
|  | 84.8 (8.0) | | 72.3 (14.8) | | | |
| BMI | 25.7 (3.0) | 29.1 (2.7) | 25.1 (0.4) | 23.2 (2.6) | 24.5 (5.1) | 25.0 (6.6) |
|  | 27.4 (3.2) | | 24.3 (3.6) | | | |
| MMSE | 29.0 (1.7) | 27.7 (1.5) | 20.5 (2.1) | 23.0 (3.0) | 20.0 (4.3) | 25.5 (0.7) |
|  | 28.3 (1.6) | | 22.1 (3.4) | | | |

Abbreviations: AD, patients with Alzheimer’s disease; EC, healthy elderly control; SD, standard deviation; 1 mg, 1 mg q.d. for 14–16 days; 0.2 mg, loading dose of 5 mg on Day 1 followed by 0.2 mg q.d. on Day 2–7; 5 mg, 5 mg q.d. for 6–9 days; 0.1 mg, loading dose of 5 mg on Day 1 followed by 0.1 mg q.d. for 13–16 days.

Supplementary Table 2

Pre-PET sembragiline plasma concentrations at steady state by dose and population

| **Dose** | **0.1 mg** | **0.2 mg** | | **1 mg** | | **5 mg** |
| --- | --- | --- | --- | --- | --- | --- |
| Population | AD | AD | EC | AD | EC | AD |
| N | 2 | 3 | 3 | 3 | 3 | 2 |
| Male, N (%) | 2 (100) | 0 | 3 (100) | 0 | 2 (66.7) | 0 |
| Mean, ng/mL | 2.55 | 7.06 | 3.29 | 41.9 | 33.9 | 96.7 |
| SD, ng/mL | 0.191 | 3.13 | 1.10 | 10.2 | 13.7 | 69.7 |

Abbreviations: AD, patients with Alzheimer’s disease; EC, healthy elderly control; SD, standard deviation; 0.1 mg, loading dose of 5 mg on Day 1 followed by 0.1 mg q.d. for Day 2–15; 0.2 mg, loading dose of 5 mg on Day 1 followed by 0.2 mg q.d. on Day 2–7; 1 mg, 1 mg q.d. for 14 days; 5 mg, 5 mg q.d. for 6–9 days.

**Supplementary Table 3**

**Injected radioactivity and total dose of the radiotracer (means (SD)) per scan and per population**

|  | **EC subjects** | | **AD patients** | | | |
| --- | --- | --- | --- | --- | --- | --- |
| Scan | 1^st^ Scan | 2^nd^ Scan | 1^st^ Scan | | 2^nd^ Scan | |
| N | 6 | 6 | 10 | | 9 | |
|  | 12 | | 19 | | | |
| Injected activity (MBq) | 293 (43) | 327 (13) | \| 271 (42) \| \| --- \| | 291 (31) | | |
|  | 310 (35) | | 280 (37) | | | |
| Total injected dose (µg) | 0.39 (0.09) | 0.18 (0.14) | 0.63 (0.81) | | | 0.47 (0.25) |
|  | 0.28 (0.16) | | 0.55 (0.60) | | | |

Abbreviations: AD, patients with Alzheimer’s disease; EC, healthy elderly control; SD, standard deviation.
